# BiPOm: a rule-based ontology to represent and infer molecule knowledge from a biological process-centered viewpoint

**DOI:** 10.1186/s12859-020-03637-9

**Published:** 2020-07-23

**Authors:** Vincent Henry, Fatiha Saïs, Olivier Inizan, Elodie Marchadier, Juliette Dibie, Anne Goelzer, Vincent Fromion

**Affiliations:** 1 Université Paris-Saclay, INRAE, MaIAGE, Jouy-en-Josas, France; 2 LRI, UMR 8623, CNRS, Université Paris-Sud, Université Paris Saclay, Orsay, France; 3 Université Paris-Saclay, INRAE, CNRS, AgroParisTech, GQE-Le Moulon, Gif-sur-Yvette, 91190 France; 4 UMR MIA-Paris, AgroParisTech, INRAE, Université Paris Saclay, Paris, France

**Keywords:** Ontology, Metabolic processes, Logical rules

## Abstract

**Background:**

Managing and organizing biological knowledge remains a major challenge, due to the complexity of living systems. Recently, systemic representations have been promising in tackling such a challenge at the whole-cell scale. In such representations, the cell is considered as a system composed of interlocked subsystems. The need is now to define a relevant formalization of the systemic description of cellular processes.

**Results:**

We introduce BiPOm (**B**iological **i**nterlocked **P**rocess **O**ntology for **m**etabolism) an ontology to represent metabolic processes as interlocked subsystems using a limited number of classes and properties. We explicitly formalized the relations between the enzyme, its activity, the substrates and the products of the reaction, as well as the active state of all involved molecules. We further showed that the information of molecules such as molecular types or molecular properties can be deduced by automatic reasoning using logical rules. The information necessary to populate BiPOm can be extracted from existing databases or existing bio-ontologies.

**Conclusion:**

BiPOm provides a formal rule-based knowledge representation to relate all cellular components together by considering the cellular system as a whole. It relies on a paradigm shift where the anchorage of knowledge is rerouted from the molecule to the biological process.

**Availability:**

BiPOm can be downloaded at *https://github.com/SysBioInra/SysOnto*

## Background

Studying and determining the characteristics of world entities and their causal relationships is at the core of many scientific endeavours, including the modern biological sciences. Managing and organizing biological data and knowledge have remained a major challenge for decades, mainly due to the high complexity of living systems. Moreover, advances of technologies in biology lead to perform high throughput experiments, that generate thousands of genes or gene products descriptions in one experiment [1]. The data associated to these experiments is growing in many application domains (e.g., plant biology, molecular biology, e-health) yielding to a bottleneck from data generation to their efficient management and the extraction of new valuable knowledge. In parallel, the systematic and intensive efforts of the biologist community greatly improve the understanding of cell functioning, particularly at the cellular and sub-cellular levels with the identification of new molecules, molecular mechanisms and their role in the whole-cell context [2, 3]. Finally, recent developments in single-cell technologies should further accentuate this general dynamic [4].

The central issue is now to find an effective and efficient way to ensure the integration of biological data originating from heterogeneous sources, while providing a formal, generic, and unambiguous representation of biological data and knowledge. For instance, let us consider the *glutamate synthase* complex *GltAB* of *Bacillus subtilis*, composed of two subunits *GltA* and *GltB*. The available information is rich [5]: we may know the structure (*GltAB* is an aggregate of four catalytic active heterodimers, consisting of a large *GltA* subunit and a small *GltB* subunit) and the cofactors (iron, iron-sulfur clusters, FAD (Flavin adenine dinucleotide), FMN (Flavin mononucleotide)). The presence of iron-sulfur clusters implies that the complex requires dedicated chaperones to be folded [6]. Although available, the ways of managing such information is far from being unified. In this example, only proteins, i.e. the subunits *GltA, GltB*, are considered and annotated but not the enzymatic complex *GltAB*, even though *GltAB* is the active compound mediating the enzymatic reaction. It may result in ambiguities, especially for the cofactors definition, and redundant information since the enzymatic complex does not exist as a cellular component by itself in repositories like Uniprot [5]. Such limitations come from the fact that the current cellular descriptions were developed together with early developments in molecular biology, where biologists were focused on gene-centered approaches. Thus, the lack of a comprehensive cell representation capturing the whole components and interactions involved in the biological process limits the benefits of the exploitation of existing knowledge and heterogeneous data.

To address this complex issue, ontologies have been widely acknowledged as a successful formal knowledge representation in many application domains including biology and many others such as geography, astronomy, medical domain and cultural heritage [7]. Indeed, ontologies provide a formal representation of entities of an application domain, called classes, and relationships between these entities, called properties. The classes are represented in a directed labeled graph structure, where the classes are linked to one to each other through semantic relations such as subsumption (e.g Tiger is-a Filadae which in turn is-a Mammal), meronymy (e.g. a finger is-a-part of a hand), equivalence and disjunction relations. Generally, the denser and deeper is the ontology graph the more specific it is.

Additional knowledge and constraints on entities and relations can be represented by domain-specific relations and/or complex logical axioms such as disjointness between classes (e.g. Oxygen is-disjoint-with Carbon), functionality and cardinality restrictions on properties (e.g. a Cell has only one Nucleus). The underlying ontology semantics is formalized as set-based logical assertions that can be either manually declared or automatically generated (i.e. inferred). Classes may have instances that must fulfill the logical axioms defined in the ontology. For instance, the statement *Tiger* is-a *Animal* expresses that all the instances of the class *Tiger* are also instances of the class *Animal*: ∀*x*:*Tiger*(*x*)⇒*Animal*(*x*). Therefore the instance Tigger of the class *Tiger* (denoted by *Tiger*(Tigger)) is an *Animal*. Moreover, to capture even more the constraints and the semantics of a given application domain, logical rules can be defined. These rules can be used by reasoners to infer new assertions from existing ones, thus enriching knowledge. For instance, the rule ∀*x*,*y*:*Animal*(*x*)∧*Animal*(*y*)∧*eats*(*x*,*y*)⇒*Carnivore*(*x*) means that every animal that eats another animal is a carnivore. Therefore, if we have *Tiger*(Tigger) ∧*Giraf*(Ernestine) ∧*eats*(Tigger, Ernestine), we can automatically deduce that Tigger is also a carnivore that is *Carnivore*(Tigger).

In biology, various bio-ontologies have been developed and stored in public repositories [8, 9]. Most of them correspond to lightweight ontologies that are suitable for specification and classification of data using annotations that link entities to their corresponding concepts in the ontology. However, these ontologies neither contain complex axioms nor rules, i.e. concepts that usually enable logical reasoning [10]. The Gene Ontology (GO) [11] is the main current ongoing initiative of the formal modelling of biological knowledge. Actually, GO describes knowledge in the domain of the molecular functions of gene products (GO-MF), cellular components (GO-CC), and biological processes (GO-BP), separately. Recently, the GO-plus project [12] integrates other bio-ontologies that were dedicated to the representation of other subcellular entities such as bio-chemicals (ChEBI; [13]) or sequence features (SO; [14]). The ontology design that is adopted in GO and GO-plus projects is to foster more specific classes and properties than generic ones, which consequently leads to a very deep hierarchy (e.g. a depth (offspring) of 16 for GO). The ongoing “Causal Activity Model” project (GO-CAM; [15]) is going one step further in expressiveness with the design of refined properties between GO-BP and GO-MF. Existing bio-ontologies are mainly used as shared controlled vocabularies between research communities setting unambiguous natural language definitions, synonyms and class annotations for biological knowledge. Therefore, bio-ontologies have been widely used to annotate biological data, in particular biological features, providing valuable inputs for many bioinformatics algorithms, such as sequence similarities for gene enrichment analysis [16] or new genome annotation [17]. Other bio-ontologies present a more generic paradigm, such as Biology Pathway Exchange (BioPAX) ontology [18], offering a language to represent pathways at the molecular and cellular level. BioPAX was not designed for annotating data but for facilitating the collection and exchange of available pathway data in a controlled way. However, the low expressiveness of current bio-ontologies limits advanced automatic reasoning for complete query answering and new information generation. In addition, the molecule function is anchored to the cellular component as an annotation whatever the state of the cellular component is. Thus, they are not fully adapted to describe the cell complexity, in particular the interplay between cellular entities and the biological processes.

Recently, the systemic description of the cell was shown to be very effective in tackling the complexity of the cell [19]. In systems biology, the description of the cell is process-centered and not gene- or molecule-centered, and is able to intrinsically handle multiple states and functions of molecules. Consequently, the function of the molecule no longer depends on the existence or on the structure of the molecule [17], but is now conditioned by the biological process to which it belongs. This paradigm shift in the description of the cell mainly corresponds to a new viewpoint that can be exploited to improve the biological processes’ description.

We previously showed in [20] that the systemic description of biological processes can be formalized as an ontological systemic model having a high level of expressiveness. As a result, the fine description of more than 200 classes of processes and sub-processes for bacterial gene-expression was related to a dozen of classes of high-level abstract processes having their own mathematical expression. Known biological processes could therefore be included in high-level classes, providing an adapted equilibrium between genericity and specificity levels. The question remains if information anchored on processes could really be used to formally describe process’ participants. Based on this previous work, we present in this article BiPOm (Biological interlocked Process Ontology for metabolism), a concise and expressive OWL-DL (Web Ontology Language) [21] ontology dedicated to the representation of metabolic processes. BiPOm can benefit from the numerous tools, such as ontology editors [22], reasoners [23], and ontology browsers [24], which are developed and widely used in the semantic Web community. BiPOm has two main original characteristics: 1) it represents biological knowledge through classes, properties, and instances and 2) it uses automatic reasoning through Semantic Web Rule Language (SWRL [25]) in order to automatically infer, formalize, and refine properties of molecules. BiPOm is an ontological model carrying the main biological processes and molecular roles/functions at a high level of abstraction where the usual annotated resources are treated as instances. As a proof-of-concept, we first apply our new ontological model to describe a complex metabolic process, the *Arabidopsis thaliana*’s “reductive pentose-phosphate cycle” (RPPC; also known as Calvin cycle), and show how properties of the cell components participating in this metabolic pathway can be automatically inferred from the formal description of a process. This example highlights how BiPOm contributes to a richer semantic description of the biological knowledge. Then, we show on a second use case that BiPOm can support the scaling up and can be instantiated with the metabolic network of the Gram negative bacterium *Escherichia coli* [26, 27].

## Results

### BiPOm overview

**Fig. 1:**
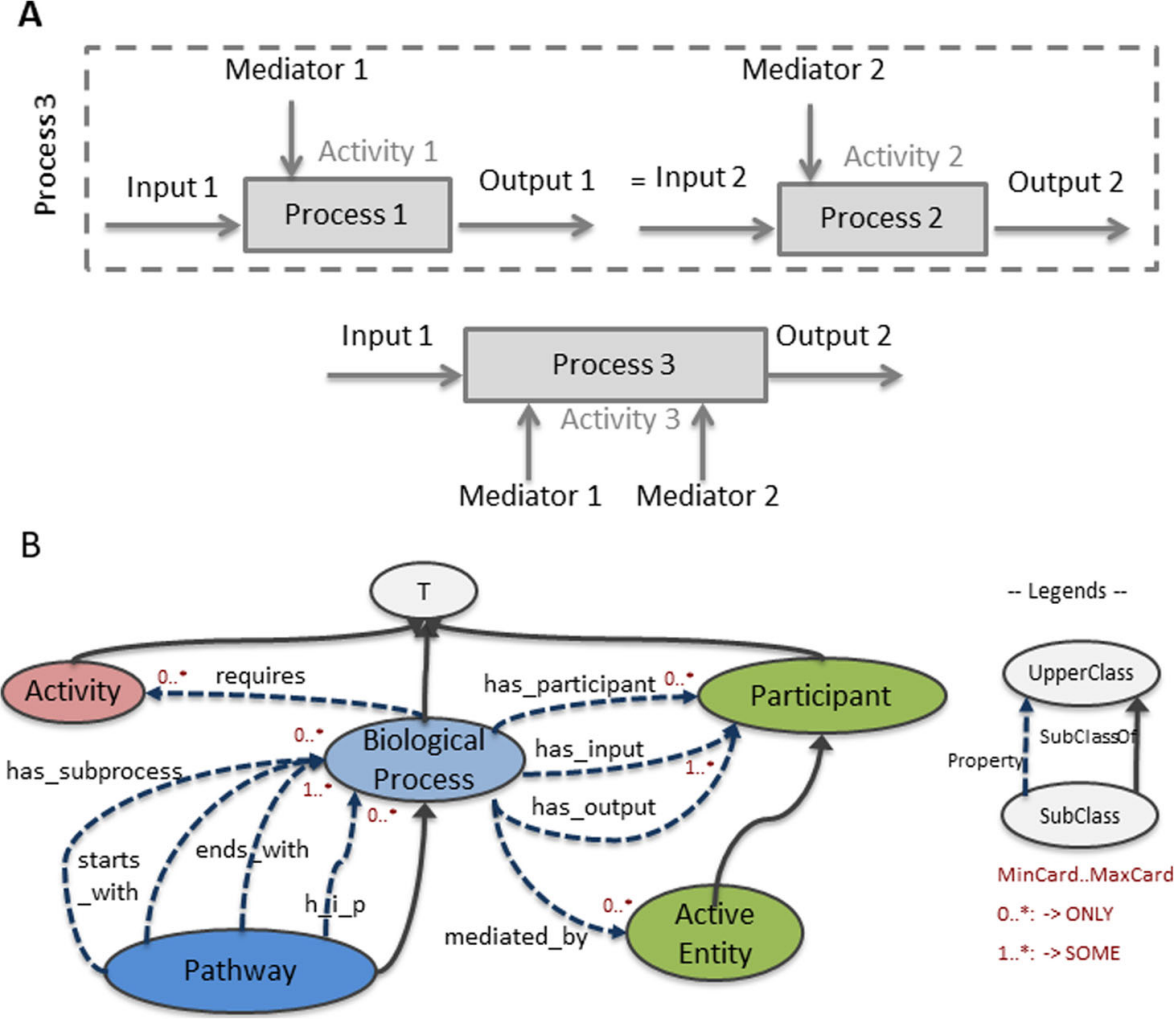
**a** Systemic representation of a metabolic pathway: two metabolic reactions (Process 1 and Process 2) that can be aggregated (Process 3). **b** Classes and properties used to formally define biological processes in BiPOm. (h_i_p: has_intermediary_process)
- molecular composition of a complex: molecule A is composed of molecules B and C
- interaction between molecules: molecule A interacts with molecule B
- molecular function of molecules: molecule A has a function of kinase activity.
- contribution of a molecule to a molecular function: molecule A contributes to kinase activity.

This work seeks to show the substantial benefits of using a concise and highly expressive (i.e. using logical axioms and rules) ontological model to describe metabolic processes (i.e. metabolic reactions, formation and activation of protein complexes) using only few knowledge of molecules. Our model was edited with Protégé [22] editor and reasoning was performed using HermiT 1.3.8 [23]. BiPOm root is divided into three disjoint main classes: biological process, participant and activity (see Fig. 1). In total, the BiPOm core-ontology contains only 141 classes. When available, the classes were imported from existing ontologies (23% from GO, 14% from Thesaurus, 13% from CHEBI; see Additional file 5: Table 2 for a complete list) and we kept the original references (such as the Internationalized Resource Identifier (IRI)) to ensure interoperability. Other classes (32%) were manually built. The classes were formally defined with 333 logical axioms using the 9 properties given in Fig. 1. Moreover, 7 inverse properties and 29 SWRL rules were defined. The logical rules infer different kind of knowledge:

- typing of molecules: for example, molecule A is a metabolite; molecule B is a kinase

### Description of the first use case: the *A. thaliana*’s reductive pentose phosphate cycle

As a use case, we considered the *A. thaliana*’s reductive pentose phosphate cycle (RPPC) available^1^ on Plant reactome [28]. This metabolic process is present in photosynthetic organisms, well described in the literature and representative of the complexity of metabolic processes. The RPPC is an essential cyclic process enabling the CO_2_ fixation and composed of 10 chemical reactions. Each chemical reaction is catalyzed by an active enzyme or enzymatic complex. The process of enzyme activation involves many different post-translational modifications, chaperoning, and complexations. In particular, the Ribulose Bisphosphate CarbOxylase (RuBisCO) enzyme that catalyzes the first reaction is an enzymatic complex composed of 16 subunits encoded by 5 genes. The activation of RuBisCO is achieved through many steps (e.g. spontaneous and chaperone-dependent complexation, carbamylation, magnesium binding, etc.) [29]. Other enzymes of the cycle are also controlled at the post-translational level by redox-reactions transfer of disulfide bonds.

### Data extraction and curation, instances definition

We collected information on the metabolic reactions and on the different steps of protein complex formation and activation using Uniprot and literature to complement the description of Plant reactome. Information was structured in a table (Additional file 5: Table 2) that was imported to instantiate BiPOm within the Protégé editor. We fully described the RPPC pathway and the RuBisCO activation pathway. RPPC pathway is an aggregated process that starts with carboxylation of D-ribulose 1,5-bisphosphate by the RuBisCO holoenzyme and ends with the phosphorylation of ribulose-5-phosphate by the reductive form of the chloroplastic Phosphoribulokinase. The RuBisCO activation pathway starts with RBCL dimerisation having RBCL as input and ends with RuBisCO-Mg complexation. The RuBisCO activation reactions involve different states of the RuBisCO: in complex with chaperones, uncarbamylated, carbamylated and associated with Mg2+ (holoenzyme). Altogether, we described 82 reactions, i.e. 24 enzymatic metabolic reactions for the RPPC and 58 post-translational protein modifications for the activation of RPPC enzymes and enzymatic complexes, 38 non-gene products and 137 gene products. The 137 gene products regroup individual proteins and protein complexes, that are primary encoded by 42 genes. The 38 non-gene products regroup the ions and metabolites involved in the 82 reactions.

For our use case, we created in BiPOm ontology 289 instances among which 123 are typed either as a biological process, a participant or an activity. We illustrated in Additional file 6: Table 3 the progressive enrichment of BiPOm content. BiPOm instantiation with RPPC information was performed with 123 declared class assertions and 469 declared property assertions. After a reasoning step using logical rules, we obtained 2163 inferred class assertions and 4218 inferred property assertions. All the 289 instances were typed by at least one BiPOm class. Moreover, the original declared class assertion during BiPOm instantiation was further specified after logical rules application. For instance, a chemical that was originally typed as GeneProduct was typed as ProteinComplexSubunit when it is part of a protein complex.

### Enrichment of knowledge on a simple enzyme

Let us illustrate the inference of properties on the phosphoglycerate kinase of *A. thaliana* that catalyzes the phosphorylation of 3-Phosphoglycerate in 1,3-Biphosphoglycerate. Information of this enzyme is available in public repositories such as Uniprot or Amigo2 (see Additional file 2: Fig. 2). Information can come from automatic or manual annotations. Automatic annotation is usually achieved by transferring known annotation (e.g. GO-Term [11]) from another organism. In BiPOm, information is provided by inferring properties of molecules using logical rules. The properties of molecules involved in the phosphoglycerate kinase are shown in Fig. 3.

The metabolic process (phosphorylation of 3PGA), the substrates (Mg-ATP, 3-Phosphoglycerate), the products (Mg-ADP, 1,3-Biphosphoglycerate), the enzyme (Phosphoglycerate kinase 1) and the enzyme activity (Phosphoglycerate kinase activity) are instances of BiPOm and are related by properties *has_input*, *has_output*, *mediated_by* and *requires*. After logical rules application, the small molecules and the enzyme are automatically typed as metabolites and kinase respectively. Moreover, the function of the enzyme (Phosphoglycerate kinase activity) is also automatically linked to the enzyme (Phosphoglycerate kinase 1) by the property *has_function*. Finally, the molecule Mg-ATP is automatically linked to its subcomponents (Mg2+ and ATP) by the property *has_molecular_part*. Compared to the static annotation given in Additional file 2: Fig. 2, the metabolic reaction is now entirely described by formal relations, that are both human- and computer-understandable.

### Enrichment of knowledge after logical rules application

In the standard gene-centered annotation, knowledge is anchored manually to protein complex subunits. Current annotation of subunits merged information on the protein itself, and on the full complex. For instance, in Amigo2, the ribulose bisphosphate carboxylase large chain (RBCL; O03042) is annotated as a magnesium dependent (magnesium(2+): CHEBI: 18420) protein (GO:0000287). This information is specific and relevant for this subunit. RBCL is further annotated 1) by the functions of the RuBisCO: Ribulose-bisphospate carboxylase activity (GO:001698) and Monooxygenase activity (GO:0004497) and 2) by the process involving RuBisCO: Reductive pentose-phosphate cycle (GO:0019253) and Photorespiration (GO:0009853) (see Fig. 4a). Such annotations should be in principle anchored to the RuBisCO holoenzyme and not to the RBCL subunit. In BiPOm, after logical rules application on instances, RBCL and Mg2+ are typed by ProteinComplexSubunit and Coenzyme, respectively (see Fig. 4b). RuBisCO holoenzyme is typed by ActiveEntity, Holoenzyme, Lyase, Oxidoreductase and ProteinComplex. Information on RuBisCO or its subunit RBCL are disjoint (see Fig. 4b): while RuBisCO holoenzyme has the function of ribulose-bisphosphate carboxylase activity, RBCL participates to the function only. We thus obtained a finer and adequate annotation of each molecular entity. In addition, we obtained information that can be computationally readable, e.g. relationship between the inputs-outputs and the reactions are formally related with the *has_input* and *has_output* properties, the protein complex and their subunits or coenzyme are formally related with the *has_molecular_part* property.

### Comparison with BioPAX

We investigated the reasoning power of BiPOm comparing to the closest work in the state of the art, that is BioPAX [18], through a quantitative comparison of both ontologies. We conducted an experimental comparison of the inferences obtained from an instantiation of Calvin Cycle (also referred to as RPPC) in BiPOm and those that are obtained in BioPAX. As we show in Additional file 7: Table 4, the number of inferred axioms is much higher in BiPOm than in BioPAX especially for property axioms. Indeed, after the reasoning, the number of property axioms has grown by 81% while in BioPAX it has grown by only 9%. Conversely to BiPOm, where many inferences are obtained thanks to the 27 logical rules, in BioPAX the inferences are mostly obtained thanks to the use of subsumption and transitivity relations.

### Scaling up to a large metabolic network: the case of *Escherichia coli*

Finally, we investigated the capability of BiPOm to describe a large metabolic network composed of more than 1500 reactions: the cytosolic metabolic network of the Gram negative bacterium *Escherichia coli*. We used the metabolic network of *Escherichia coli*, because of (*i*) the accuracy of the metabolic reconstruction, (*ii*) the quality of annotations and cross-references of reactions, metabolites and gene products [26, 30], and (*iii*) the description of enzymatic complexes (including cofactor recruitment and subunit structures), all of this information being available in standardized files [27, 31]. The final use case is composed of 1576 cytosolic metabolic reactions, 1027 metabolites, 1109 enzymatic complexes whose subunits are encoded by 1441 genes, and 1766 additional reactions to describe the formation of active enzymatic complexes. We then generated the instantiation table (Additional file 8: Table 5) based only on the available information [26, 27, 30], that was imported to instantiate BiPOm within the Protégé editor. We illustrated in Additional file 9: Table 6 the progressive enrichment of BiPOm content. BiPOm instantiation with *E. coli* information was performed with 10055 instances (3653 declared class assertions and 16093 declared property assertions). After a reasoning step using logical rules, we obtained 71990 inferred class assertions and 82774 inferred property assertions. This example highlights that automatic reasoning on a large metabolic network can be achieved while allowing a substantial enrichment of the initial knowledge.

## Discussion

Here we introduced BiPOm, a concise and highly expressive ontology describing metabolic processes. We explicitly handled the different states of molecules as instances including the “active state” involved in a metabolic process, and more generally in a biological process, which are traditionally poorly described in bio-ontologies. Using SWRL rules to perform automatic reasoning on BiPOm instances, we enriched information on cellular entities such as molecular types or molecular properties from a few assertions used to describe biological processes. Enriched knowledge is encoded in a formal language. We thus made explicit and machine-readable some implicit biological knowledge. Few information is required to instantiate BiPOm and can be extracted from existing databases or bio-ontologies and completed (if necessary) by information from literature. Our approach takes advantage of existing public repositories to finely describe biological processes and benefits from the ontological reasoning to infer new types and properties. The use case of *A. thaliana*’s RPPC is representative of the complexity of metabolic processes and highlights the added value of BiPOm representation. Thanks to BiPOm rules, the classical annotations stored in public repositories such as Amigo2 or Uniprot were automatically inferred as new properties. By dealing with different aggregation levels of biological processes, BiPOm can take into account heterogeneous levels of knowledge on biological pathways. Moreover, BiPOm includes GeneProductModificationProcess classes that are able to describe post-translational modification processes. Thus, in addition to metabolic pathways, BiPOm is already capable of describing a large set of non-metabolic processes involving post-translational modifications of proteins and protein complexes. In particular, post-translational modifications are commonly encountered in signalling pathways or cascades processes (see for example the descriptions of the RuBisCO activation pathway or the Glyceraldehyde-3-phosphate dehydrogenase regulation by oxido-reduction in Additional file 5: Table 2). Finally, we showed that BiPOm is able to describe the cytosolic metabolic network of *Escherichia coli*. The use of BiPOm for any organism could be limited by the availability of information, i.e. the complete description of enzymatic reactions, of catalyzing enzymatic complexes and of their formation. However, with the advent of whole-cell models, the information starts to be available for bacteria [27, 32] and should become more and more available in the future, even for multi-cellular organisms [33].

Due to its flexibility, the extension of BiPOm to the subcellular localization of components should be straightforward since different localizations of a molecule can be managed as different states. This requires to integrate additional classes for the fine description of subcellular compartments. However, enriching the ontology with precise information of molecule localization may be difficult, because the localization of molecules may not be well characterized experimentally, especially for eukaryotic cells. In addition, our ontology could be extended with other biological processes such as the gene-expression [34] using the same ontological model. Only the class hierarchy has to be extended with new subclasses such as a subclass of the class Participant representing the sequence patterns [14] or a subclass of the class Activity representing the RNA polymerase activity. Finally, according to our previous works [34], each biological process could be linked by a formal property to a mathematical model that describes the kinetic behavior of the process. This could be achieved using a few additional properties and classes from the Bacterial interlocked Process ONtology (BiPON) [34].

There exist other attempts of biological process formalism, and especially BioPAX [18]. BioPAX aims at sharing knowledge on metabolic pathways and reactions using an ontological model. By doing so, BioPAX bypasses the heterogeneity of formalisms of public repositories that describe metabolic pathways such as WikiPathways [35], KEGG [36] or Reactome [37] and increases interoperability between them. The underlying ontological model contains properties describing molecular- and process-centered relationships, but is less expressive than BiPOm. To fairly compare the two ontologies, we performed automatic reasoning on the same use case, the RPPC. The final number of property axioms was 166% more important in BiPOm than BioPAX, thus revealing the interest of using a richer semantics for describing the metabolic pathways. This comparison shows that if biological ontologies like BioPAX were enriched by logical rules and high level axioms as done in BiPOm, they will both reduce the number of subsumption relations and properties that must be declared in the ontology and empower the inference task in biological ontologies [38].

Recent releases of knowledge-based repositories such as Reactome [37] or disease maps [39] are now based on systemic representations and use System Biology Mark-up Language (SBML; [31]) to describe reactions. They may provide an adequate framework to integrate the diversity of knowledge and heterogeneous data (e.g. genomics, transcriptomics, post-translational modifications of proteins). However, the SBML formalism is currently not expressive enough to provide insurance in consistency and constraints satisfiability as an ontological model does. A framework converting SBML models into a OWL format exists [40]. It aims at improving the annotation of SBML but without revisiting the formal description of metabolic process. However, such a framework could be used to assist BiPOm’s instantiation.

## Conclusion

In conclusion, BiPOm is based on a minimal and generic ontological model and aims to take maximum advantage of automatic reasoning to limit the number of assertions and enrich initial knowledge. Logical reasoning has already been exploited by other bio-ontologies mainly for the automatic classification of molecules [41, 42] or phenotypes [43]. Here we provide not only the automatic classification of biological process and molecules, but also inferences of semantics relationships based on asserted axioms. Moreover, while other works on automatic reasoning needs computing extension to the ontological model [44], our ontology is autonomous. This is due to the combination of the instantiation of classes (compared to usual annotation), the use of SWRL rules (bypass of open-world assumption) and the designed knowledge model itself (systemic representation is a graph by itself). The knowledge enrichment in BiPOm is also due to the process-centered representation of cells, and more deeply to the systemic nature of biological objects. If this concept emerged almost 20 years ago (see for instance [45]), it can only now be formalized into axiomatically rich ontologies. Indeed, such a type of formalization requires a sufficiently global understanding of the object’s functioning, so that a high level of genericity of their formal description can be achieved. With the advent of the first whole-cell systemic models [32, 46], we know that the level of knowledge is now sufficient to tackle the formalization of the cell functioning. A first step was achieved in previous works [34] and now in this paper. BiPOm provides a formal rule-based knowledge representation to relate all cellular components together by considering the cellular system as a whole.

## Methods

**Notation:** For the sake of readability of this section, we use the following notation. Names of classes are written in lower-case and first letter capitalized (e.g., GeneProduct for the class named "Gene Product”) and properties are written in lower case and in italics (e.g. *has_input* refers to the property named “has_input").

### A concise and expressive formal representation of metabolic reactions and pathways

In systems biology, the cell is considered as a system composed of interlocked subsystems having their own dynamics of operations. In this approach, a subsystem is a biological process (and its related biological subprocesses). A process can be independently defined as (see Fig. 1a):

- **Elementary** by its inputs and outputs and possibly by a specific mediator and/or by an activity;
- **Aggregated** by a set of elementary processes.

Furthermore, a metabolic reaction is defined as an elementary biological process having molecular entities in inputs (i.e. the reactants) and in outputs (i.e. the products) (Fig. 1a). A metabolic reaction also has a metabolic activity corresponding to the type of the chemical reaction (e.g. hydrolysis, kinase). The metabolic reaction can be spontaneous or mediated by an active cell component such as an enzyme or a protein complex. A succession of metabolic reactions defines a metabolic pathway [36]. A metabolic pathway is thus represented as an aggregated process composed of several successive elementary processes.

This representation relies on a limited set of classes, cardinalities and properties presented in Fig. 1b. Briefly, the classes BiologicalProcess (in blue on Fig. 1b), Activity (in red), Participant, and ActiveEntity (in green) contain the metabolic reactions, the metabolic activities, the molecules and the active cell components respectively. Since an active cell component is a molecular entity, the class ActiveEntity is by definition a subclass of Participant. The property *has_participant* and its subproperties *has_input*, *has_ouput*, *mediated_by* define the relations between the classes BiologicalProcess, Participant, and ActiveEntity. The property *requires* defines the relation between the class BiologicalProcess and the class Activity. Finally, the class Pathway is defined by successive biological processes. The first, last and intermediate biological processes within a pathway are set up using the *has_subprocess* subproperties *start_with*, *end_with* and *has_intermediary_process* respectively. We further detail in the next subsections the BiPOm core-ontology classes, properties and rules.

### Classes of BiPOm core-ontology

#### The biologicalProcess class

**Fig. 2:**
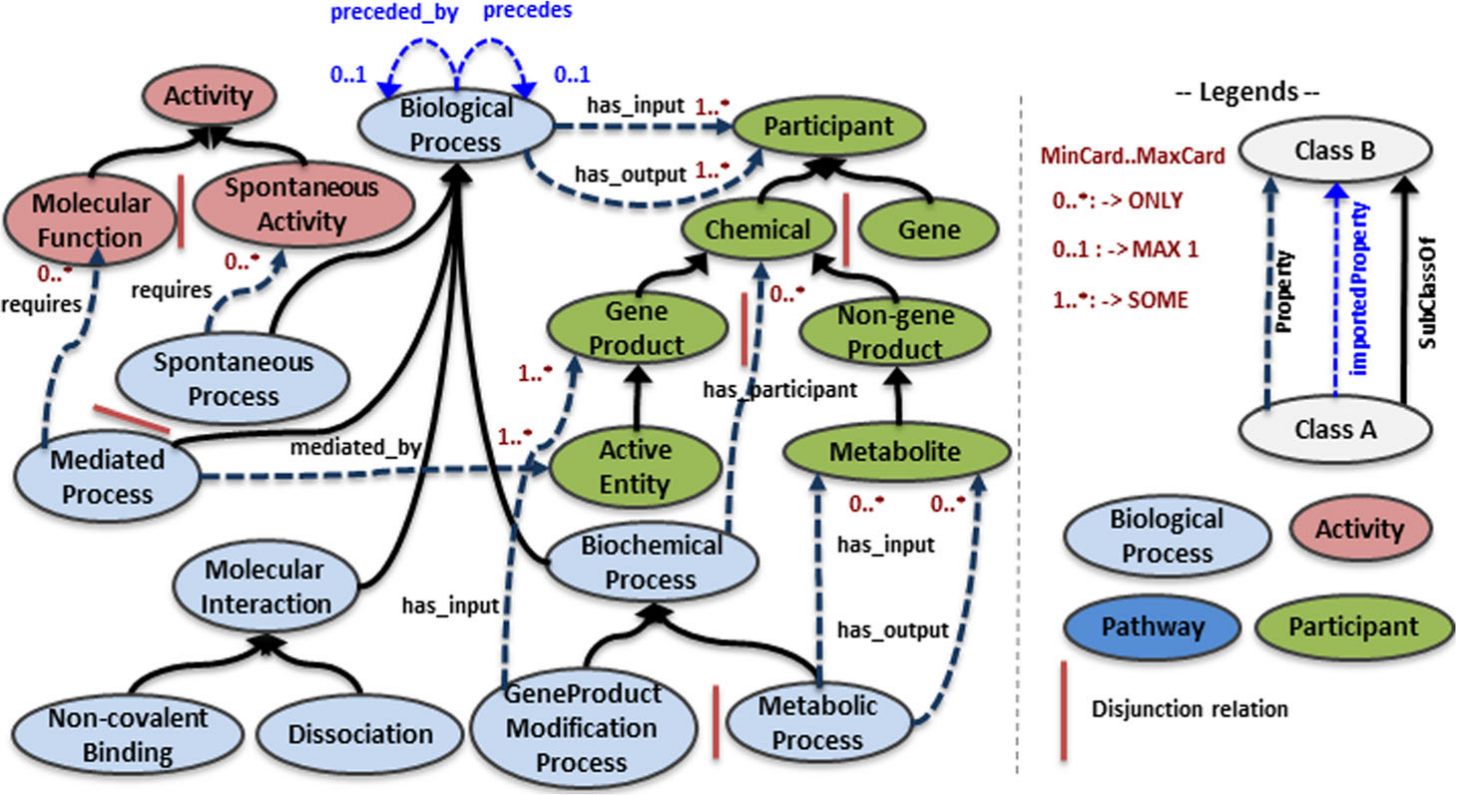
High-level classes and properties of BiPOm core-ontology

Our aim is to easily represent any biological process involved in metabolic networks. By definition, such processes gather the metabolic biochemical reactions and the post-translational processes involved in the activation of molecular machineries, protein complexes or enzymes of the metabolic network. We described the subclasses of BiologicalProcess (see blue classes on Fig. 2) that were organized according to (a) the type of the process (e.g. biochemical process, molecular interaction), (b) the type and number of the molecular entities involved in the process (e.g. metabolites only, a combination of proteins and metabolites), (c) the fact that a process can occur spontaneously or be driven by an active molecular entity.

**Fig. 3:**
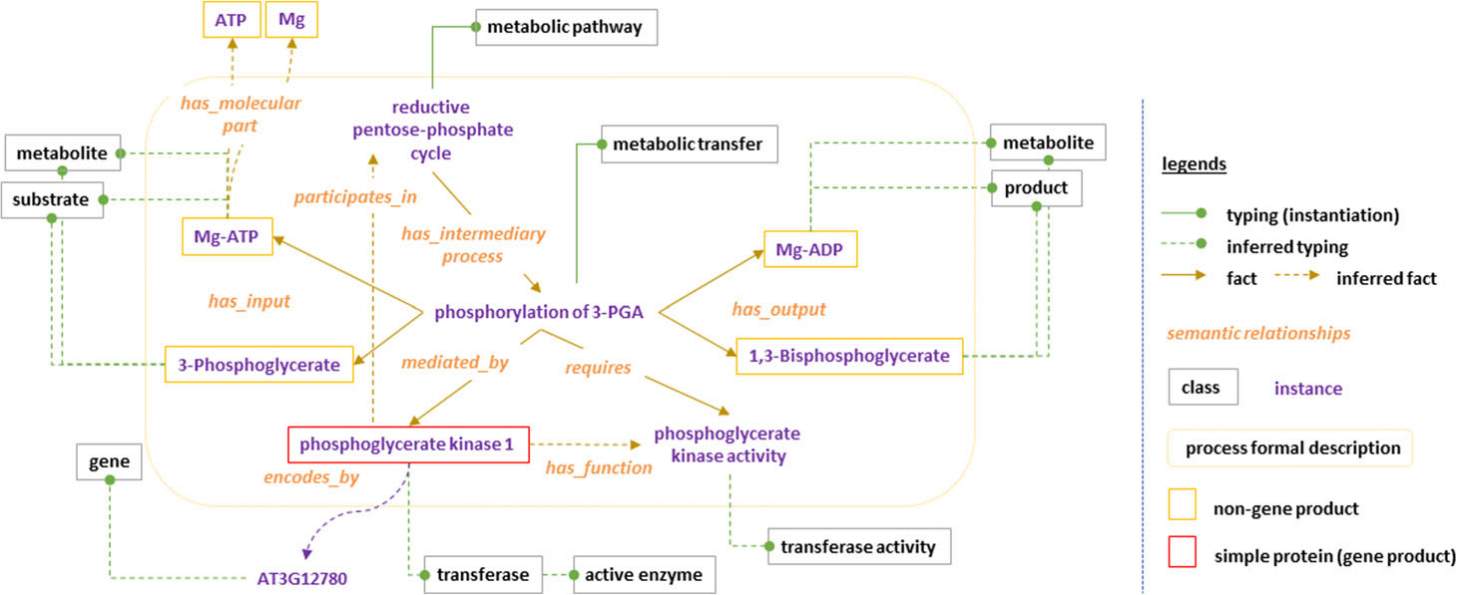
Formal description of the instance *phosphoglycerate kinase* of *A. thaliana* in BiPOm

**Fig. 4:**
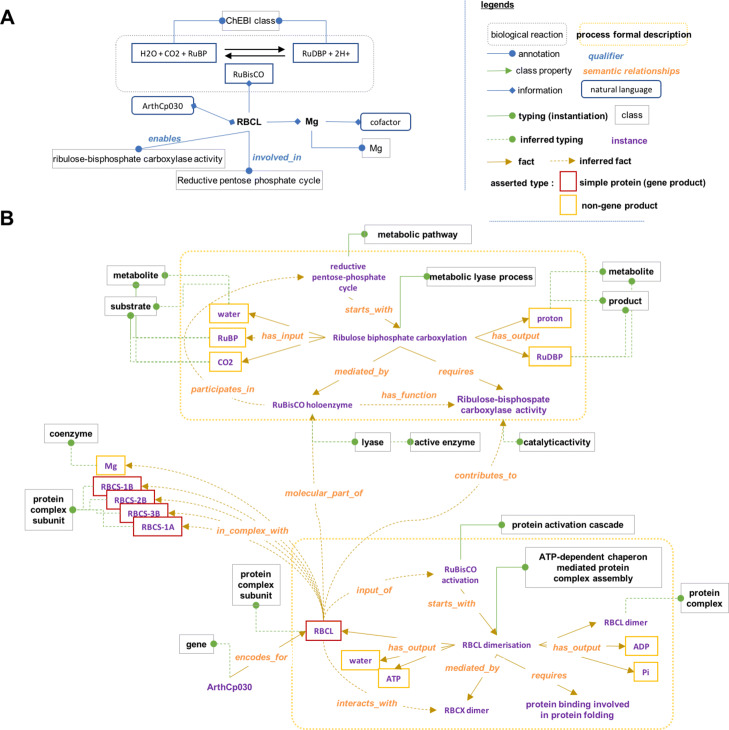
Information on RuBisCO large subunit supported by ontological resources: (**a**) Annotation in Uniprot or AmiGO2, (**b**) formal relation using BiPOm. (RAF1: Rubisco accumulation factor 1; RBCL: Ribulose bisphosphate carboxylase large chain; RBCX; Chloroplastic Chaperonin-like RBCX protein. GP: gene-product; NGP: non-gene product)

The class BiologicalProcess is decomposed into four subclasses: BiochemicalProcess, MolecularInteraction, and the two disjoint subclasses SpontaneousProcess or MediatedProcess. Using axioms we define that any instance of the subclass BiologicalMediatedProcess will be mediated at least by one active molecular entity. The class BiochemicalProcess is further decomposed into two disjoint classes MetabolicProcess and GeneProductModificationProcess. A metabolic process is defined (using axioms) as a metabolic reaction having only metabolites in inputs and outputs. In contrast, a gene product modification process is defined such that it has at least one gene product as input of the process. Finally, the class MolecularInteraction is decomposed into the two disjoint classes Non-covalentBinding and Dissociation, to describe molecule association and dissociation.

Then, specific biological processes are defined according to the role of their participants. Two examples of description are given in Additional file 1: Fig. 1: an enzymatic metabolic reaction (see Additional file 1: Fig. 1A, which is a subclass of MetabolicProcess and EnzymaticReaction) and a protein complex assembly (see Additional file 1: Fig. 1B), which is a a subclass of SpontaneousProcess, Non-covalentBinding and Post-translationalProteinModification.

Altogether our model contains 65 processes’ classes with a maximum depth of 7.

#### The participant class

The class Participant describes the entities that are inputs, outputs or mediators of biological processes (see green classes on Fig. 2). The entity can be a chemical (subclass Chemical) or a sequence (subclass Gene). A chemical can further be a gene-product such as a protein or a protein complex (subclass GeneProduct) or a biochemical (subclass Non-geneProduct). Subclasses Chemical/Gene and GeneProduct/Non-geneProduct are disjoint. GeneProduct corresponds to gene-dependent macromolecules that are usually annotated by GO [11]. On the other hand, Non-geneProduct refers to biochemicals that are not specific to an organism and typically belong to ChEBI classes [12]. Types of biochemicals (e.g. cofactor, metabolite, ions) and of gene-products (e.g. enzyme) are respectively defined as subclasses of Non-geneProduct and GeneProduct. For instance, Metabolite is a subclass of Non-geneProduct (see Fig. 1a).

#### The activity class

The class Activity contains activities of spontaneous and mediated processes such as hydrolysis activity, i.e. the hydrolysis of a chemical bond (see red classes on Fig. 2). The class Activity is divided into two disjoint subclasses, MolecularFunction and SpontaneousActivity that correspond to the activities of mediated and spontaneous processes respectively. The class MolecularFunction includes GO-MF classes related to catalytic activity or chaperoning activity. The class SpontaneousActivity includes GO-MF classes related to binding activities.

### BiPOm rules and properties

According to Fig. 2, BiPOm core-ontology contains 28 properties. Among them, 7 properties were built to be explicitly asserted in the ontology: *has_input*, *has_output*, *mediated_by*, *requires*, *starts_with*, *ends_with*, and *has_intermediary_process* (see A:1-7 in Additional file 4: Table 1). Other properties can be automatically inferred using automatic reasoning thanks to 10 inverse properties definitions (see IP:1-10 in Additional file 4: Table 1) and to 27 SWRL (Semantic Web Rule Language) rules definition (see R:1-27 in Additional file 4: Table 1). Notice that a same property can be inferred using different SWRL rules (i.e. *has_molecular_part*). Moreover, some SWRL rules infer both properties as well as instance typing, i.e. to which class an instance is belonging to (see R:7-13 in Additional file 4: Table 1).

Let us now detail four of the main SWRL rules defined for inferring four kinds of different molecule properties: *has_function* that links formally the biological process activity to the active molecular entity, *has_molecular_part* that links formally the subunits or the co-enzyme to the complex, *contributes_to* that links formally the complex’s subunits to the complex’s function and finally *interacts_with* that links formally the complex’s subunits or the biological process’s inputs with the process’s mediator.

***has_function:*** the molecular function of an active gene product is assigned to the activity of the biological process by the *has_function* property. In natural language, *has_function* is expressed as:“If a biological mediated reaction *r* which required *a* as activity is mediated by the participant *p*_0_, then *p*_0_*has_functiona*." This expression is translated in the SWRL rule *R*_27_ presented in Additional file 4: Table 1 and illustrated in Fig. 3 defined as follows:

$$\begin{array}{ll} R_{27}: & \texttt{BiologicalMediatedReaction}(\mathrm{r}) \wedge \texttt{BiologicalProcess}(\mathrm{p}_{0}) \wedge \\ &\hspace{0cm} \texttt{Activity}(\mathrm{a}) \wedge {mediated\_by}(\mathrm{r},\mathrm{p}_{0}) \wedge {requires}(\mathrm{r},\mathrm{a}) \\ [3pt] &\hspace{4cm} \Rightarrow {has\_function}(\mathrm{p}_{0},\mathrm{a}) \end{array} $$

A biological process is usually mediated by an active molecular entity such as a protein complex that can be composed especially of several subunits and co-enzymes. In BiPOm, further information is inferred from the components of the active molecular entity and more particularly how each component is defined as a subpart of the complex, how it contributes to the function of the complex and what are its interactions. This is achieved with the three following rules:

***has_molecular_part:*** the assembly of a protein complex can be spontaneous, be assisted by a chaperone, or be ATP-dependent which results in several SWRL rules for the property ***has_molecular_part*** (see Additional file 4: Table 1). Here we present the case where the protein complex assembly is an ATP-dependent process, which produces ADP and P. If a process *proc* of type ProteinComplexAssembly has for output a participant *p*_0_ that differs from ADP or phosphate (P), and if *proc* has also for input a simple protein *p*_*i*_, then *p*_0_*has_molecular_part* the participant *p*_*i*_, and *p*_*i*_ is a ProteinComplexSubunit. This can be expressed in the SWRL rule *R*_10_ presented in Additional file 4: Table 1 and illustrated in Fig. 4 defined as follows:

$$\begin{array}{ll} R_{10}: & \texttt{ProteinComplexAssembly}(\text{proc}) \wedge \cdots \\ &{has\_output}(\text{proc},\mathrm{p}_{0}) \wedge \text{DifferentFrom}(\text{ADP},\mathrm{p}_{0}) \wedge \cdots\\ & \text{DifferentFrom}(\mathrm{P},\mathrm{p}_{0}) \wedge {has\_input}(\text{proc},\mathrm{p}_{\mathrm{i}}) \wedge \cdots\\ & \wedge \texttt{SimpleProtein}(\mathrm{p}_{\mathrm{i}}) \cdots\\ & \hspace{1.5cm} \Rightarrow {has\_molecular\_part}(\mathrm{p}_{0},\mathrm{p}_{\mathrm{i}}) \wedge \cdots \wedge\\ & \hspace{2cm} \texttt{ProteinComplexSubunit}(\mathrm{p}_{\mathrm{i}}) \end{array} $$

***contributes_to:*** let us assume that the participant *p*_0_ mediates the reaction *r* which requires *a* as activity, and that *p*_0_ is composed of several molecular parts. Then, each molecular part of *p*_0_*contributes_to* the activity *a*. This can be expressed in the SWRL rule *R*_26_ presented in Additional file 4: Table 1 and illustrated in Fig. 4 defined as follows:

$$\begin{array}{ll} R_{26}: & {has\_function}(\mathrm{p}_{0},\mathrm{a}) \wedge {molecular\_part\_of}(\mathrm{p}_{0},\mathrm{p}_{\mathrm{i}}) \\ & \hspace{2cm}\Rightarrow {contributes\_to}(\mathrm{p}_{\mathrm{i}},\mathrm{a}) \end{array} $$

***interacts_with:*** using SWRL rules, we designed properties that define transient interaction between proteins. The property *interacts_with* is particularly used to describe processes of post-translational modifications: If a process *proc* has a protein *prot* as an input and is mediated by another participant *p*_0_ then *protinteracts_withp*_0_. This can be expressed in the SWRL rule *R*_25_ presented in Additional file 4: Table 1 and illustrated in Fig. 4 defined as follows:

$$\begin{array}{rl} R_{25}:& \texttt{Protein}(\text{prot}) \wedge {has\_input}(\text{proc},\text{prot}) \wedge \cdots \\ &\hspace{0.0cm} {mediated\_by}(\text{proc}, \mathrm{p}_{0}) \wedge \text{DifferentFrom}(\text{prot}, \mathrm{p}_{0}) \\ &\hspace{0.6cm} \Rightarrow {interacts\_with}(\text{prot},\mathrm{p}_{0}) \end{array} $$

Finally, we imported some properties from the ontology BiPON [34] (e.g. *precedes* and its inverse property *preceded_by*) to provide information on the relative order of biological processes. They are able to order reactions in a pathway. The properties *precedes* and *preceded_by* are used to infer participants of a pathway, while excluding those that are produced and consumed by consecutive reactions (see Additional file 4: Table 1).

### Limited amount of information for instantiation

Thanks to logical axioms and rules, only few declared assertions are necessary to instantiate BiPOm in order to represent the data and knowledge of a metabolic pathway (e.g. the *A. thaliana*’s reductive pentose phosphate cycle (RPPC) or the metabolic network of *E. coli*). Briefly, each instance of processes of the studied application domain has to be typed by one class among the 65 different processes’ classes in BiPOm. Each instantiated process is then described by highlighting its links with the instances of its inputs, its outputs, its mediators (if any) and its activity through *has_input*, *has_output*, *mediated_by* and *requires* properties, respectively. The whole pathways can therefore be described by their links to the starting, intermediate and ending processes through the *starts_with*, *has_intermediary_process* and *ends_with* properties, respectively. The instances of the participants have to be typed by the class Gene, GeneProduct or Non-geneProduct. The manual typing of an instance by a class is called a *declared class assertion*. The manual assertion of a property between two instances is called a *declared property assertion* hereafter. For example, the biological process "phosphorylation of 3-PGA" and the metabolite "Mg-ATP" are typed manually by the class MetabolicTransference and the class Non-geneProduct respectively (and thus correspond to *declared property assertions*). In addition, we specified manually the declared property assertion that phosphorylation of 3-PGA *has_input* Mg-ATP (see Additional file 5: Table 2).

Information on instances could be easily structured in a table (see Additional file 3: Text 1, Additional file 5 and 8) and imported in BiPOm using the cellfie plugin [47]. Then, the HermiT reasoner automatically infers additional information (called *inferred -class or -property assertions*) from the instances [48]. The BiPOm core-ontology and its instantiation after logical rules application are available at *https://github.com/SysBioInra/SysOnto*. The different steps of instantiation and reasoning are summarized in Additional Table 3 for the RPPC of *A. thaliana* and in Additional Table 6 for the metabolic network of *E. coli*.

## Supplementary information

**Additional file 1** Examples of description of biological processes by BiPOm. Two biological processes (A) an enzymatic metabolic reaction and B) a protein complex assembly) are described by classes and properties of BiPOm.

**Additional file 2** The phosphoglycerate kinase of *A. thaliana*. Available information on the phosphoglycerate kinase of *A. thaliana* in public repositories such as Uniprot or Amigo2

**Additional file 3** Guidelines. This additional file aims at assisting the creation of the instantiation table for BiPOm feeding, such as Additional Table 5.

**Additional file 4** List of BiPOm properties. Additional Table 1 contains the properties of BiPOm that can be (i) asserted, (ii) defined as the inverse of an asserted property, or (iii) defined by a SWRL rule.

**Additional file 5** Table of BiPOm instantiation for the rPPC of *Arabidopsis thaliana*. Additional Table 2 contains the instances of BiPOm classes necessary to describe the RPPC of *A. thaliana*.

**Additional file 6** Metrics of BiPOm before and after reasoning on the rPPC of *A. thaliana*. Additional Table 3 contains the metrics of BiPOm (i) before and (ii) after instantiation, and finally (iii) after automatic reasoning.

**Additional file 7** Quantitative comparison of inferences in BiPOm and in bioPAX. Additional Table 4 contains the number of class/property axioms before and after reasoning in both BiPOm and BioPAX.

**Additional file 8** Table of BiPOm instantiation for the metabolic network of *Escherichia coli*. Additional Table 5 contains the instances of BiPOm classes necessary to describe the metabolic network of *E. coli*.

**Additional file 9** Metrics of BiPOm before and after reasoning on the metabolic network of *E. coli*. Additional Table 6 contains the metrics of BiPOm (i) before and (ii) after instantiation, and finally (iii) after automatic reasoning.

## Data Availability

All versions of BiPOm can be downloaded at *https://github.com/SysBioInra/SysOnto*: (i) BiPOm-core ontology, (ii) BiPOm after instantiation with RPPC and *E. coli* information and (iii) BiPOm after automatic reasoning. Moreover, BiPOm-core can also be downloaded from Bioportal at *http://bioportal.bioontology.org/ontologies/BIPOM*.

## Abbreviations

BiPOm: Biological interlocked Process Ontology for metabolism
FAD: Flavin adenine dinucleotide
FMN: Flavin mononucleotide
GO: Gene Ontology
GO-MF: GO-molecular functions
GO-CC: GO-cellular components
GO-BP: GO-Biological Processes
SO: Sequence Ontology
GO-CAM: GO-Causal Activity Model
ChEBI: Chemical Entities of Biological Interest
BioPAX: Biology Pathway Exchange
OWL-DL: Web Ontology Language
SWRL: Semantic Web Rule Language
RPPC: Reductive Pentose-Phosphate Cycle
IRI: Internationalized Resource Identifier
RuBisCO: Ribulose Bisphosphate CarbOxylase
RBCL: Ribulose bisphosphate carboxylase large chain
3PGA: 3-phosphoglycerate
ATP: Adenosine triphosphate
ADP: Adenosine diphosphate
P: Phosphate
Mg: Magnesium
RNA: ribonucleic acid
BIPON: Bacterial interlocked Process ONtology
SBML: System Biology Markup Language

## Footnotes

1 http://plantreactome.gramene.org/PathwayBrowser/#/R-ATH-1119519&SEL=R-ATH-5149661&PATH=R-ATH-2744345,R-ATH-2883407&DTAB=MT

## References

[1] Mayer B. Bioinformatics for omics data: methods and protocols. 2011. n ^∘^57: 004 BIO.

[2] Brown A, Fernández IS, Gordiyenko Y, Ramakrishnan V (2016). Ribosome-dependent activation of stringent control. Nature.

[3] Mets FD, Melderen LV, Gottesman S (2018). Regulation of acetate metabolism and coordination with the TCA cycle via a processed small RNA. Proc Nat Acad Sci.

[4] Baron M, Yanai I (2017). New skin for the old RNA-Seq ceremony: the age of single-cell multi-omics. Genome Biol.

[5] Consortium U (2014). UniProt: a hub for protein information. Nucleic Acids Res.

[6] Johnson DC, Dean DR, Smith AD, Johnson MK (2005). Structure, function, and formation of biological iron-sulfur clusters. Annu Rev Biochem.

[7] Staab S, Studer R (2009). Handbook on Ontologies. 2nd ed.

[8] Whetzel PL, Noy NF, Shah NH, Alexander PR, Nyulas C, Tudorache T (2011). BioPortal: enhanced functionality via new Web services from the National Center for Biomedical Ontology to access and use ontologies in software applications. Nucleic Acids Res.

[9] Smith B, Ashburner M, Rosse C, Bard J, Bug W, Ceusters W (2007). The OBO Foundry: coordinated evolution of ontologies to support biomedical data integration. Nat Biotech.

[10] Van Heijst G, Schreiber AT, Wielinga BJ (1997). Using explicit ontologies in KBS development. Int J Hum Comput Stud.

[11] Consortium TGO (2004). The Gene Ontology (GO) database and informatics resource. Nucleic Acids Res.

[12] Hill DP, Adams N, Bada M, Batchelor C, Berardini TZ, Dietze H (2013). Dovetailing biology and chemistry: integrating the Gene Ontology with the ChEBI chemical ontology. BMC Genomics.

[13] Hastings J, de Matos P, Dekker A, Ennis M, Harsha B, Kale N (2013). The ChEBI reference database and ontology for biologically relevant chemistry: enhancements for 2013. Nucleic Acids Res.

[14] Eilbeck K, Lewis SE, Mungall CJ, Yandell M, Stein L, Durbin R (2005). The Sequence Ontology: a tool for the unification of genome annotations. Genome Biol.

[15] Consortium TGO (2017). Expansion of the Gene Ontology knowledgebase and resources. Nucleic Acids Res.

[16] Martin D, Brun C, Remy E, Mouren P, Thieffry D, Jacq B (2004). GOToolBox: functional analysis of gene datasets based on Gene Ontology. Genome Biol.

[17] Balakrishnan R, Harris MA, Huntley R, Van Auken K, Cherry JM. A guide to best practices for Gene Ontology (GO) manual annotation. Database. 2013. 10.1093/database/bat054. 10.1093/database/bat054 PMC3706743 23842463

[18] Demir E, Cary MP, Paley S, Fukuda K, Lemer C, Vastrik I (2010). The BioPAX community standard for pathway data sharing. Nat Biotechnol.

[19] Kildegaard HF, Baycin-Hizal D, Lewis NE, Betenbaugh MJ (2013). The emerging CHO systems biology era: harnessing the ’omics revolution for biotechnology. Curr Opin Biotechnol.

[20] Henry V, Ferré A, Froidevaux C, Goelzer A, Fromion V, Boulakia SC, et al.Représentation systémique multi-échelle des processus biologiques de la bactérie. In: IC 2016 : 27ème Journées francophones d’Ingénierie des Connaissances (Proceedings of the 27th French Knowledge Engineering Conference), Montpellier, France, June 6-10, 2016: 2016. p. 97–102. http://hal.archives-ouvertes.fr/IC_2016/hal-01442727.

[21] Bechhofer S, van Harmelen F, Hendler J, Horrocks I, McGuinness DL, Patel-Schneider PF, et al.OWL Web Ontology Language Reference. W3C. 2004. http://www.w3.org/TR/owl-ref/.

[22] Musen MA (2015). The protégé project: a look back and a look forward. AI Matters.

[23] Glimm B, Horrocks I, Motik B, Stoilos G, Wang Z (2014). HermiT: An OWL 2 Reasoner. J Autom Reason.

[24] Lohmann S, Negru S, Haag F, Ertl T (2016). Visualizing Ontologies with VOWL. Semant Web.

[25] Horrocks I, Patel-Schneider PF, Boley H, Tabet S, Grosofand B, Dean M. SWRL: A Semantic Web Rule Language Combining OWL and RuleML. 2004. http://www.w3.org/Submission/SWRL/.

[26] Orth JD, Conrad TM, Na J, Lerman JA, Nam H, Feist AM (2011). A comprehensive genome-scale reconstruction of Escherichia coli metabolism—2011. Mol Syst Biol.

[27] Bulović A, Fischer S, Dinh M, Golib F, Liebermeister W, Poirier C (2019). Automated generation of bacterial resource allocation models. Metab Eng.

[28] Naithani S, Preece J, D’Eustachio P, Gupta P, Amarasinghe V, Dharmawardhana PD (2017). Plant Reactome: a resource for plant pathways and comparative analysis. Nucleic Acids Res.

[29] Andersson I, Backlund A (2008). Structure and function of Rubisco. Plant Physiol Biochem.

[30] King ZA, Lu J, Dräger A, Miller P, Federowicz S, Lerman JA (2016). BiGG Models: A platform for integrating, standardizing and sharing genome-scale models. Nucleic Acids Res.

[31] Smith LP, Hucka M, Hoops S, Finney A, Ginkel M, Myers CJ (2015). SBML level 3 package: Hierarchical model composition, version 1 release 3. J Integr Bioinformatics.

[32] Karr JR, Sanghvi JC, Macklin DN, Gutschow MV, Jacobs JM, Bolival Jr B (2012). A whole-cell computational model predicts phenotype from genotype. Cell.

[33] Goelzer A, Fromion V (2017). Resource allocation in living organisms. Biochem Soc Trans.

[34] Henry VJ, Goelzer A, Ferré A, Fischer S, Dinh M, Loux V (2017). The bacterial interlocked process ONtology (BiPON): a systemic multi-scale unified representation of biological processes in prokaryotes. J Biomed Semant.

[35] Slenter DN, Kutmon M, Hanspers K, Riutta A, Windsor J, Nunes N (2017). WikiPathways: a multifaceted pathway database bridging metabolomics to other omics research. Nucleic Acids Res.

[36] Kanehisa M, Furumichi M, Tanabe M, Sato Y, Morishima K (2016). KEGG: new perspectives on genomes, pathways, diseases and drugs. Nucleic Acids Res.

[37] Fabregat A, Jupe S, Matthews L, Sidiropoulos K, Gillespie M, Garapati P (2017). The reactome pathway knowledgebase. Nucleic Acids Res.

[38] Cheung KH, Qi P, Tuck D, Krauthammer M (2006). A semantic web approach to biological pathway data reasoning and integration. J Web Semant.

[39] Mazein A, Ostaszewski M, Kuperstein I, Watterson S, Le Novère N, Lefaudeux D (2018). Systems medicine disease maps: community-driven comprehensive representation of disease mechanisms. NPJ Syst Biol Appl.

[40] Lister AL, Lord P, Pocock M, Wipat A (2010). Annotation of SBML models through rule-based semantic integration. J Biomed Semant.

[41] Wolstencroft K, Lord P, Tabernero L, Brass A, Stevens R (2006). Protein classification using ontology classification. Bioinformatics.

[42] Magka D, Krötzsch M, Horrocks I (2014). A rule-based ontological framework for the classification of molecules. J Biomed Semant.

[43] Mungall CJ, Gkoutos GV, Smith CL, Haendel MA, Lewis SE, Ashburner M (2010). Integrating phenotype ontologies across multiple species. Genome Biol.

[44] Blondé W, Mironov V, Venkatesan A, Antezana E, De Baets B, Kuiper M (2011). Reasoning with bio-ontologies: using relational closure rules to enable practical querying. Bioinformatics.

[45] Kitano H (2001). Foundations of Systems Biology.

[46] Goelzer A, Muntel J, Chubukov V, Jules M, Prestel E, Nölker R (2015). Quantitative prediction of genome-wide resource allocation in bacteria. Metab Eng.

[47] Kola JS, Rector A. Importing Spreadsheet data into Protegé́: Spreadsheet plug-in. In: Proc. of the Intl. Protégé Conference: 2007. https://protege.stanford.edu/conference/2007/presentations/10.03_Kola.pdf.

[48] Glimm B, Horrocks I, Motik B, Stoilos G, Wang Z (2014). HermiT: An OWL 2 Reasoner. J Autom Reason.

