# Supplementary figures and images for: BiPOm: a rule-based ontology to represent and infer molecule knowledge from a biological process-centered viewpoint
Source: BMC Bioinformatics. 2020 Jul 23;21:327. doi: 10.1186/s12859-020-03637-9 (PMC7376860; doi:10.1186/s12859-020-03637-9)

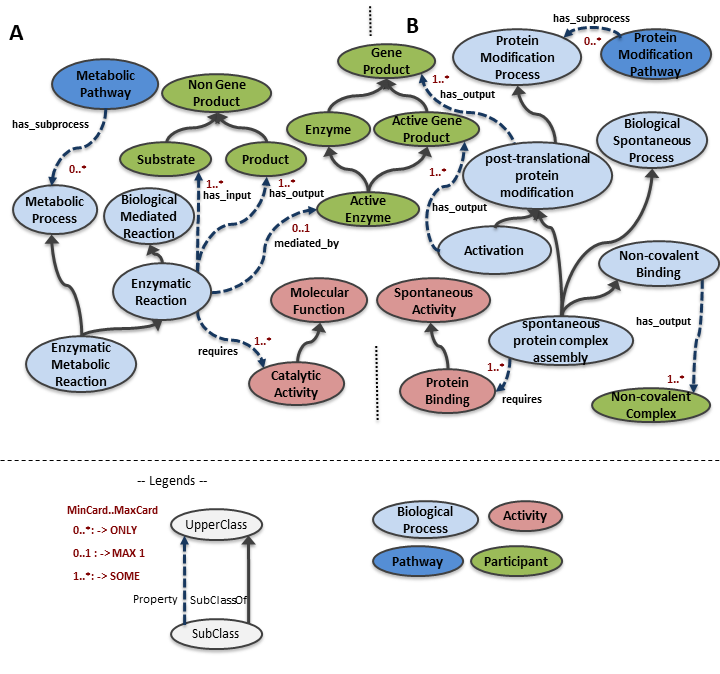

Supplement: Supplementary file 1 — Additional file 1 Examples of description of biological processes by BiPOm. Two biological processes (A) an enzymatic metabolic reaction and B) a protein complex assembly) are described by classes and properties of BiPOm. [file 12859_2020_3637_MOESM1_ESM.png]

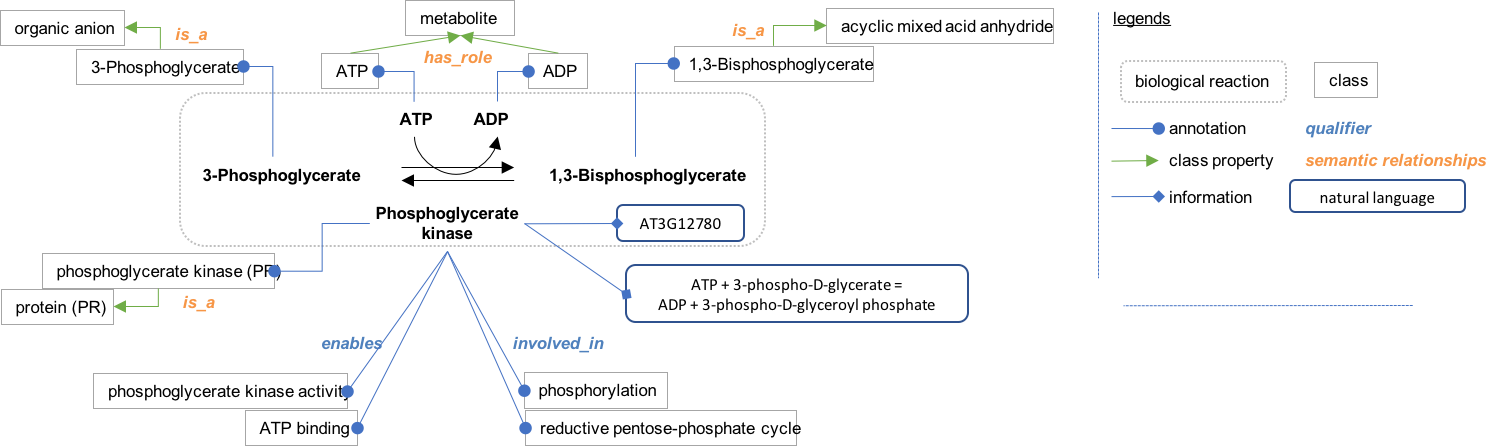

Supplement: Supplementary file 2 — Additional file 2 The phosphoglycerate kinase of A. thaliana. Available information on the phosphoglycerate kinase of A. thaliana in public repositories such as Uniprot or Amigo2 [file 12859_2020_3637_MOESM2_ESM.png]
