# Supplementary material for: BiPOm: a rule-based ontology to represent and infer molecule knowledge from a biological process-centered viewpoint
Source: BMC Bioinformatics. 2020 Jul 23;21:327. doi: 10.1186/s12859-020-03637-9 (PMC7376860; doi:10.1186/s12859-020-03637-9)
Supplement: Supplementary file 3 — Additional file 3 Guidelines. This additional file aims at assisting the creation of the instantiation table for BiPOm feeding, such as Additional Table 5. [file 12859_2020_3637_MOESM3_ESM.docx]

# Guidelines for BiPOm feeding

This document provides a guideline for BiPOm use and feeding.

## I. Information extraction and curation

The information necessary to feed BiPOm corresponds to the metabolic reactions, that can be grouped in metabolic pathways, the description of enzymatic complexes, and of the formation of enzymatic complexes.

### I.1 The metabolic reactions and pathways

Extract from a public repository (i.e. Reactome, KEGG, MetaCyc) the metabolic pathways for the organism of interest. Alternatively, the metabolic pathway (or reactions) can be extracted from a so-called genome-scale metabolic model (if it exists) for the organism of interest. Such models are usually available in a standardized XML format called SBML (Hucka et al. Bioinformatics 2003 , 19(4): 524-531)). Last releases of SBML models (Orth et al. Molecular systems biology 2011, 7:1) contain cross-references such as CheBI identifiers for metabolites, Rhea, KEGG or BioCyc identifiers for reactions, which makes them a reliable and centralized source of information.

### I.2 The formation of enzymatic complexes

Determine the active form of each enzymatic complex. Some of them are described in public repositories such as Reactome, KhloroKB, or disease maps. For bacteria, resource allocation models can also provide a description in a standardized format (Bulovic et al. Metabic Engineering 2019, 55: 12-22). Otherwise, the active form of complexes should be determined from the literature. The active form of the enzymatic complex may include several steps, such as homo- or heteromer steps of protein complexation, cofactor recruitment or post-translational modications (e.g. phosphorylation), chaperoning to cite a few. Such type of information may be found in Uniprot, but is usually anchored on each protein subunit. In that case, manual curation may be necessary to determine precisely the composition and formation of the enzymatic complexes.

## II. Formalization using BiPOm

The information is summarized in a Table used to instantiate BiPOm. The table contains three excel sheets :

1. **process**: that contains the metabolic reactions, the pathway definition, and the reactions involved in the formation of active enzymatic complexes
2. **chemical non-gene products** : that contains the metabolites
3. **chemical gene and gene products** : that contains the genes, and the native native form of polypeptidic chains involved in the metabolic reactions.

### II.1 Process

(i) Describe the metabolic reactions : identify the inputs, the outputs, and the mediator if any, and the activity.

Below is the table for the metabolic reaction :

ATP-Mg + 3-phospho-D-glyceric acid → ADP-Mg + 3-phospho-D-glyceroyl dihydrogen phosphate

This reaction is mediated by the Phosphoglycerate kinase 1.

| **Class assertion** | **Process** | **Input** | **Output** | **Mediator** | **Activity** | **Cross references (of Activity)** |
| --- | --- | --- | --- | --- | --- | --- |
| Metabolic transference | phosphorylation of 3-PGA | ATP-Mg | ADP-Mg | Phospho-glycerate kinase 1 | Phospho- glycerate kinase activity | http://purl.obolibrary.org/obo/GO_0004618 |
|  | phosphorylation of 3-PGA | 3-phospho-D-glyceric acid | 3-phospho-D-glyceroyl dihydrogen phosphate |  |  |  |

When the reaction has several inputs and/or several outputs, the table has several lines (as many lines as the maximum of inputs/outputs). The name of the process should be repeated each time, because the cellfie plugin will always link the instance of inputs (or outputs) to the « Process » instance of the same line.

(ii) Describe the formation of active enzymatic complexes by generating as many as intermediate complexes as necessary. Each complex must be either the result of a process (i.e. is the output of a process), either a native protein present in the sheet  « **chemical gene and gene products »**.

(iii) Define a metabolic pathway by describing the first, last and intermediate metabolic processes :

| **Class Assertion** | **Superprocess** | **starts_with** | **ends_with** | **intermediary_process** |
| --- | --- | --- | --- | --- |
| Metabolic pathway | reductive pentose-phosphate cycle | Ribulose bisphosphate carboxylation | ribulose 5-phosphate phosphorylation | Ribose 5-phosphate - Xylulose 5-phosphate production-Ca |
|  | reductive pentose-phosphate cycle |  |  | sedoheptulose-1,7-bisphosphate aldolisation |
|  | ... |  |  | ... |

The instances of processes in the columns **starts_with, ends_with** and **intermediary_process** must also be defined in the column « Process ». The name of the metabolic pathway should be repeated several times, because the cellfie plugin will always link the instance of the starting, ending or intermediate process to the « Superprocess »’s instance of the same line. Moreover, it takes as many lines as there are intermediate processes in a metabolic pathway.

### II.2 Chemical non-gene products

### List the metabolites involved in all reactions, and create a table with four columns : (i) BiPOm class assertion (i.e. NonGene-Product or Ion), (ii) Identifier of the metabolites, (iii) Name of the metabolite and (iv) the CheBI IRI for cross-references.

### The instances in the column «Identifier» must be the same as those present in the Process sheet as inputs/outputs of a process.

### **Mandatory Identifiers:** Some SWRL rules involve ATP, phosphate, ADP, H2O and H+. The identifiers of these metabolites must be **ATP**, **phosphate(3-)**, **ADP**, **water** and **proton** respectively in the sheets **Process** and **Chemical non-gene products**. This will ensure the proper functioning of SWRL rules.

### Each individual metabolite involved a reaction must be declared in the sheet **Chemical non-gene products.**

### II.3 Chemical gene and gene products

List all genes and native protein names that are present in the metabolic reactions and build a table of five columns : (i) Gene identifier (e.g. b2519), (ii) Gene name (e.g. *ndk*), (iii) Uniprot URL, (iv) Uniprot identifier (e.g. P0A763 for *ndk*), (v) Native protein name (from Uniprot, e.g Nucleoside diphosphate kinase).

### Each native protein involved a reaction must be declared in the sheet **Chemical gene and gene products.**

### III. Instantiation and inference using Protege

Install Protege and the Cellfie plugin on your computer.

Use the Cellfie plugin to import the table of instantiation.

Run automatic reasoning with HermiT and edit the inferences. All molecules and their different states are now typed by a class. Their semantics is enriched with new information (other than the asserted ones, i.e. *has_input*, *has_output*, *mediated_by, requires, starts_with, ends_with, has_intermediary_process).*
